# The anatomic analysis of the vidian canal and the surrounding structures concerning vidian neurectomy using computed tomography scans^☆^

**DOI:** 10.1016/j.bjorl.2017.11.008

**Published:** 2017-12-26

**Authors:** Gülay Açar, Aynur Emine Çiçekcibaşı, İbrahim Çukurova, Kemal Emre Özen, Muzaffer Şeker, İbrahim Güler

**Affiliations:** a Necmettin Erbakan University, Meram Faculty of Medicine, Department of Anatomy, Konya, Turkey; b Health Sciences University, Izmir Tepecik Trainig and Research Hospital, Department of Otolaryngology-Head and Neck Surgery, Izmir, Turkey; c Katip Çelebi University, Faculty of Medicine, Department of Anatomy, Izmir, Turkey; d Selcuk University, Faculty of Medicine, Department of Radiology, Konya, Turkey

**Keywords:** Intrasphenoid septum, Morphometric analysis, Pterygoid process pneumatization, Vidian canal, Vidian neurectomy, Septo intraesfenoidal, Análise morfométrica, Pneumatização do processo pterigoide, Canal pterigoideo, Neurectomia do pterigoideo

## Abstract

**Introduction:**

The type of endoscopic approach chosen for vidian neurectomy can be specified by evaluating the vidian canal and the surrounding sphenoid sinus structures.

**Objective:**

The variations and morphometry of the vidian canal were investigated, focusing on the functional correlations between them which are crucial anatomical landmarks for preoperative planning.

**Methods:**

This study was performed using paranasal multidetector computed tomography images that were obtained with a section thickening of 0.625 mm of 250 adults.

**Results:**

The distributions of 500 vidian canal variants were categorized as follows; Type 1, within the sphenoid corpus (55.6%); Type 2, partially protruding into the sphenoid sinus (34.8%); Type 3, within the sphenoid sinus (9.6%). The pneumatization of the pterygoid process is mostly seen in vidian canal Type 2 (72.4%) and Type 3 (95.8%) (*p* < 0.001). The mean distances from the vidian canal to the foramen rotundum and the palatovaginal canal were greater in the vidian canal Type 2 and 3 with the pterygoid process pneumatization (*p* < 0.001). The prevalence of the intrasphenoid septum between the vidian canal and the vomerine crest and lateral attachment which ending on carotid prominence were much higher in vidian canal Type 3 than other types (*p* < 0.001). The mean angle between the posterior end of the middle turbinate and the lateral margin of the anterior opening of the vidian canal was measured as 33.05 ± 7.71°.

**Conclusions:**

Preoperative radiologic analysis of the vidian canal and the surrounding structures will allow surgeons to choose an appropriate endoscopic approach to ensure predictable postoperative outcomes.

## Resumo

**Introdução:**

O tipo de abordagem endoscópica para a neurectomia do vidiano pode ser definido pela avaliação do canal do vidiano e das estruturas adjacentes aos seios esfenoidais.

**Objetivo:**

Investigar as variações e a morfometria do canal vidiano com enfoque nas suas correlações funcionais, pois são parâmetros anatômicos cruciais para o planejamento pré-operatório.

**Método:**

Esse estudo foi realizado utilizando-se imagens de tomografia computadorizada multidetectores dos seios paranasais com espessura de corte de 0,625 mm obtidas de 250 indivíduos adultos.

**Resultados:**

A distribuição das 500 variantes do canal vidiano foi categorizada da seguinte forma: Tipo 1, dentro do corpo ósseo esfenoidal (55,6%); Tipo 2, protrusão parcial no interior do seio esfenoidal (34,8%); Tipo 3, no interior do seio esfenoidal (9,6%). A pneumatização do processo pterigoide foi observada principalmente no canal vidiano Tipo 2 (72,4%) e Tipo 3 (95,8%) (*p* < 0,001). As distâncias médias do canal vidiano até o forame redondo e o canal palatovaginal foram maiores no canal vidiano do Tipo 2 e 3, com a pneumatização do processo pterigoide (*p* < 0,001). A presença do septo intraesfenoidal entre o canal vidiano e a crista vomeriana e a extensão lateral, que termina na proeminência da carótida, foi muito maior no canal vidiano Tipo 3 do que nos outros tipos (*p* < 0,001). A angulação média entre a cauda da concha média e a margem lateral da abertura anterior do canal vidiano foi de 33,05° ± 7,71°.

**Conclusões:**

A análise radiológica pré-operatória do canal do vidiano e das estruturas circunjacentes permitem ao cirurgião escolher uma abordagem endoscópica apropriada e prever resultados pós-operatórios.

## Introduction

The pterygopalatine fossa (PPF) looks like an inverse pyramid in a relatively deep inaccessible anatomical location that is formed by the perpendicular lamina of palatine bone medially, greater wing of sphenoid bone superiorly, the pterygoid process of sphenoid bone posteriorly and maxillary sinus anteriorly.^1^ The various osseous communications of the PPF forms recognizable anatomical landmarks on Multidetector Computed Tomography (MDCT) scans that describe the margins and openings of the PPF.^2^ From lateral to medial the foramen rotundum (FR), the vidian canal (VC) which is located inferomedial to FR and the palatovaginal canal (PVC) are clinically important openings including critical neurovascular structures in the posterior wall of the PPF. Also, these foramina are in close association with the surrounding sphenoid sinus structures.^1^

The endoscopic surgery to resect a tumor that localizes in or around the PPF and to cure the chronic vasomotor rhinitis is an alternative to traditional surgical approach and minimizes morbidity and the size of the incision.^3^ Currently, endoscopic transnasal and transsphenoid vidian neurectomy are performed; the success rate of both approaches can be influenced by the different surrounding sphenoid structure variations such as; VC protrusion into the sphenoid sinus, intrasphenoid septum, the pterygoid process pneumatization (PPP) and the relationship of the VC with the middle nasal turbinate.^4^ So, preoperative radiological evaluation of the VC corpus types and surrounding sphenoid structures guides the surgeon in choosing an appropriate surgical approach, decreasing complications during endoscopic surgery.^5^

In this study, we observed VC variations and studied the morphometric parameters of the VC related to the surrounding sphenoid structures. Specifically, we focused on the relationship between them and analyzed the factors which affect the formation of various VC corpus types relating to endoscopic approaches.

## Methods

The approval of this retrospective investigation was done by our local Ethics Committee with an approval number 2016/543 and performed using paranasal MDCT images of 250 patients who were referred to Department of Radiology from January 2016 to July 2016. All patients were evaluated using 128 slice MDCT scanner (Siemens, imaging parameters: kV, 120; mA, 160; rotation time, 0.5 s; collimation, 128 × 0.625; FOV, 220 mm). Multiplanar reconstruction images (associated coronal and sagittal images of 1 mm slice thickness) were generated on the basis of the axial images which were obtained with a section thickening of 0.625 mm. According to a premade protocol on Syngo Via (Siemens, Germany) all scans were analyzed. The patients with a previous paranasal sinus surgery, nasal trauma or fractures, and paranasal tumors were excluded.

**Figure 1:**
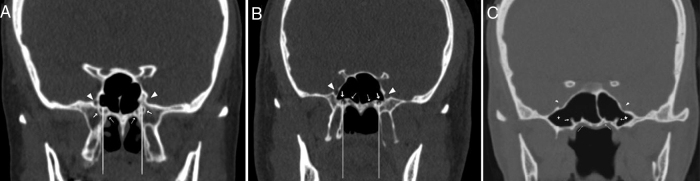
Coronal CT sections showing the vidian canal (thick arrow), palatovaginal canal (thin arrow), foramen rotundum (arrowhead), pterygoid recess pneumatization (star). (A) Bilateral vidian canals inside the sphenoid corpus (VC Type 1), the right vidian canal located lateral to medial pterygoid plate and left located at same line. (B) The vidian canals which partially protruding into the sphenoid sinus (VC Type 2) bilaterally located at same line with the medial pterygoid plate. (C) The vidian canals which totally inside the sphenoid sinus with a stalk (VC Type 3).

**Figure 2:**
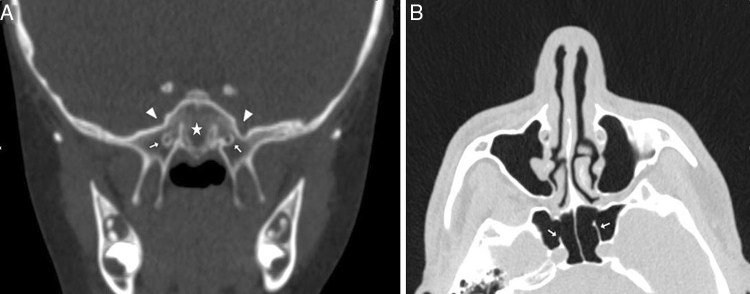
(A) Coronal CT section showing the conchal type sphenoid pneumatization pattern (star); (B) Axial CT section showing the intrasphenoid septum which deviated and attached on the carotid prominence (ICAS) (thick arrow).

**Figure 3:**
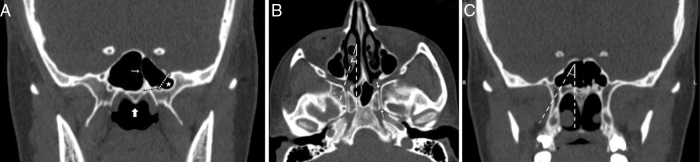
Morphometric measurements (A) Coronal CT sections showing the distance from the vidian canal to palatovaginal canal (black dotted line) and the foramen rotundum (white dotted line), the septum (thin arrow) between the vidian canal and vomerine crest (thick arrow), the pterygoid process pneumatization (star). (B) Axial CT section showing the angle between the posterior end of the middle turbinate and lateral margin of the anterior opening of the vidian canal, the anterior opening of the vidian canal (thick arrow) and the posterior opening of the vidian canal (thin arrow). (C) Coronal CT sections showing the angle between the posterior end of the middle turbinate and lateral margin of the anterior opening of the vidian canal.

We observed the variations of the VC corpus types and the surrounding structures as shown in Table 1. The VC corpus types according to the relationship with the sphenoid sinus were categorized into three types which are based on Yeh et al.’s^4^ classification as follows; Type 1, within the sphenoid corpus (Fig. 1A); Type 2, partially protruding into the sphenoid sinus (Fig. 1B); Type 3, totally protruding into the sphenoid sinus with a stalk (Fig. 1C). The localization of VC according to the medial plate of the pterygoid process (MPP) was defined as medial (including medial and same line) and lateral (Fig. 1A and B). The sphenoid pneumatization pattern (Fig. 2A), the intrasphenoid septum between the VC and the vomerine crest (Fig. 1A and C) and lateral attachments of the septum which ends on the carotid prominence (ICAS) (Fig. 2B) or the optic canal (OCS) were analyzed. Also, the presence of the PPP, which was defined as the sphenoid pneumatization extended into the pterygoid process was observed (Figure 1, Figure 3). The nasal septum deviation was determined as to right, to left and no septum deviation.

**Table 1:** Definitions of variations of the vidian canal and the surrounding structures.

Variations	Definitions
*VC corpus types*	
Type 1	The vidian canal was located within sphenoid bony roof
Type 2	The vidian canal was partially protruded into sphenoid sinus
Type 3	The vidian canal was totally protruded into sphenoid sinus with a stalk

*Intrasphenoid septum types*	
Vomerine crest-VC	The sphenoid septum between the vomerine crest and the vidian canal
ICAS	The sphenoid septum which deviated laterally and attached to the carotid prominence
OCS	The sphenoid septum which deviated laterally and attached to the optic canal

*Pterygoid process pneumatization*	
Existing	The sphenoid pneumatization extended into the pterygoid process
Not existing	There was no pneumatization in the pterygoid process

*VC-medial plate of the pterygoid process relationship*	
Medial	The vidian canal was located medially to the medial plate of the pterygoid process or on same line
Lateral	The vidian canal was located laterally to the medial plate of the pterygoid process

*Nasal septum deviation*	
Right	The nasal septum deviating to right
Left	The nasal septum deviating to left
Absent	There was no septum deviation

**Figure 4:**
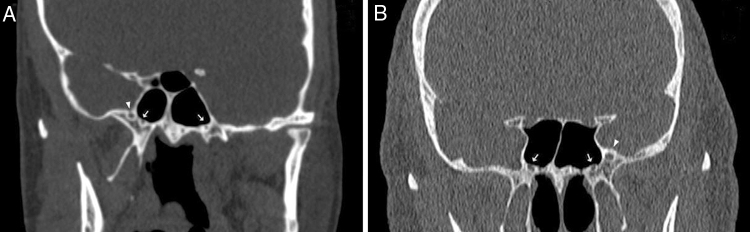
(A) Coronal CT image of 19-year-old woman showing unilateral right foramen rotundum (arrowhead, she had no left foramen rotundum) and the vidian canals (thick arrow); (B) coronal CT image of 68-year-old man showing unilateral left foramen rotundum (arrowhead, he had no right foramen rotundum) and the vidian canals (thick arrow).

As seen in Table 2, the morphometric parameters including the mean diameter of the sphenopalatine foramen (SPF), the distances from the vomerine crest to the PVC and VC, the distance between the FR and the VC were measured in coronal plane (Fig. 3A). To predict the feasibility of vidian neurectomy, we measured the mean angle between the posterior end of the middle turbinate (MTP) and lateral margin of the anterior opening of the VC (Fig. 3B and C). Also, the length and the mean diameters of the middle part, anterior and posterior openings of the VC were measured in axial plane (Fig. 3C). In our study, a female patient who was 19-years-old had no left FR (Fig. 4A) and a male patient who was 68-years-old had no right FR (Fig. 4B).

**Table 2:** Definitions of the measurements.

Measurements	Definitions
*VC diameters*	
Anterior	The anterior opening of the vidian canal on posterior wall of the pterygopalatine fossa
Middle	The middle part of the vidian canal
Posterior	The posterior opening of the vidian canal on anterior margin of the foramen lacerum
VC length (A–P)	The distance from the anterior to posterior openings of the vidian canal

*VC location*	
PVC-vomerine crest	The distance from the palatovaginal canal to the vomerine crest
VC-vomerine crest	The distance from the vidian canal to the vomerine crest
VC-FR	The distance from the vidian canal to the foramen rotundum

*SPF diameter*	
Posterior to the middle turbinate	The diameter between the perpendicular plate of the palatine bone (inferior) and the sphenoid bone (superior)

*Endoscopic endonasal approach angle*	
VC-MTP Angle	The angle between the posterior end of the middle turbinate and the most lateral margin of the anterior opening of the vidian canal

SPSS 22 (SPSS, Inc., Chicago, IL, USA) was used for statistical analysis. For statistical comparisons, Chi-square test, unpaired *t*-test, One-Way Analysis of Variance (ANOVA) were used and a value of *p* < 0.05 was considered significant.

## Results

In this study, the patients consisted of 107 females (43%) and 143 males (57%) with a median age of 45.76 ± 17.64 years for females and 40.80 ± 16.47 years for males. Three variations of VC corpus anatomy according to the relationship with the sphenoid sinus were found as follows: Type 1 which was located within sphenoid bony roof (55.6%, 278/500), Type 2 which was partially protruded into sphenoid sinus (34.8%, 174/500), Type 3 which was totally protruded into sphenoid sinus with a stalk (9.6%, 48/500). We determined that the number of the intrasphenoid septum with single septa was 197 (78.8%), with multiple septa was 45 (18%), and without septa was 8 (3.2%). The distribution ratios of the sphenoid septum types were found as follows: intrasphenoid septum between VC and the vomerine crest as 13.2% (66/500), lateral attachment of the septum which ends on the carotid prominence (ICAS) as 28.4% (142/500) and on the optic canal (OCS) as 14.4% (72/500). Also, we found the pattern of the sphenoid pneumatization as the sellar type (67%) and the prevalence of the presence of the PPP as 39.2% (196/500). The VC mostly located medial to the MPP (82%, 410/500) and the prevalences of the septum deviation were found as to right 34.4% (86/250), to left 25.2% (63/250), no septum deviation 40.4% (101/250). The PVC was identified in 372 of the 500 computed tomography (CT) coronal sections (74.4%). The relationship of the VC corpus types with the surrounding structures variations is shown in Table 3. The ratios of the presence of the PPP, the ICAS, the septum between VC and vomerine crest were significantly higher in VC Type 2 and 3 than Type 1 (*p* < 0.001, *p* = 0.03). Also, the concurrence of the ICAS and the PPP was statistically significant (*p* < 0.001), as seen in Table 4.

**Table 3:** The relationship between the variations of the vidian canal types and the surrounding structures.

The surrounding structure variations	VC type			*p*-Value
	Type 1 N° (%)	Type 2 N° (%)	Type 3 N° (%)	
*SS related with ICA*				
Not existing	223 (80.2%)	105 (60.3%)	30 (62.5%)	<0.001
Existing	55 (19.8%)	69 (39.7%)	18 (37.5%)	

*Pterygoid pneumatization*				
Not existing	254 (91.4%)	48 (27.6%)	2 (4.2%)	<0.001
Existing	24 (8.6%)	126 (72.4%)	46 (95.8%)	

*SS related with OC*				
Not existing	237 (85.3%)	148 (85.1%)	43 (89.6%)	0.71
Existing	41 (14.7%)	26 (14.9%)	5 (10.4%)	

*SS between VC and Vomerine crest*				
Not existing	251 (90.3%)	145 (83.3%)	38 (79.2%)	0.03
Existing	27 (9.7%)	29 (16.7%)	10 (20.8%)	

*VC relation with MPP*				
Medial	219 (78.8%)	153 (87.9%)	38 (79.2%)	0.06
Lateral	59 (21.2%)	21 (12.1%)	10 (20.8%)	

VC, Vidian canal; Type 1, within sphenoid corpus; Type 2, partially protruding into the sphenoid sinus; Type 3, totally protruding into the sphenoid sinus; SS, sphenoid septum; ICA, internal carotid artery; OC, optic canal; MPP, medial pterygoid plate.

**Table 4:** The relationship between the pterygoid pneumatization and the surrounding structures variations.

The surrounding structures variations	Pterygoid process pneumatization		*p*-Value
	Not existing N° (%)	Existing N° (%)	
*SS related with ICA*			
Not existing	234 (77.0%)	124 (63.3%)	<0.001
Existing	70 (23.0%)	72 (36.7%)	

*SS related with OC*			
Not existing	257 (84.5%)	171 (87.2%)	0.40
Existing	47 (15.5%)	25 (12.8%)	

*SS between VC and vomerine crest*			
Not existing	271 (89.1%)	163 (83.2%)	0.05
Existing	33 (10.9%)	33 (16.8%)	

*VC relation with MPP*			
Medial	333 (78.0%)	477 (88.3%)	0.06
Lateral	67 (22.0%)	23 (11.7%)	

SS, sphenoid septum; ICA, internal carotid artery; OC, optic canal; VC, vidian canal; MPP, medial pterygoid plate.

We measured the mean diameters of the middle part, anterior and posterior openings of the VC as 1.0 ± 0.4 mm, 1.9 ± 0.6 mm and 1.6 ± 0.5 mm, respectively. Also, the mean length of the VC was found as 12.9 ± 1.9 mm. The mean distances from the vomerine crest to VC and to PVC were measured as 13.6 ± 1.7 mm and 5.9 ± 3.9 mm, respectively. The mean distance between VC and FR was 8.1 ± 2.3 mm, and the mean diameter of the SPF was 3.9 ± 0.8 mm. The mean value for the angle between the MTP and lateral margin of the anterior opening of the VC was 33.05 ± 7.71°. Table 5, Table 6 summarize the relationship of the morphometric measurements with the VC variations and the PPP. According to Table 5, Table 6 the VC Type 3 and the PPP altered the VC-FR and VC-PVC distances as increased, but the opposite was true for the PVC-vomerine crest distance, the anterior and middle diameters of the VC that were smaller in VC Type 3 and PPP (*p* < 0.001).

**Table 5:** The relationship between the morphometric measurements and the vidian canal corpus types.

Morphometric measurements	Total	VC type			*p*-Value
		Type 1	Type 2	Type 3	
VCP diameter (mm)	1.6 ± 0.5	1.6 ± 0.5	1.6 ± 0.5	1.5 ± 0.5	0.25
VCM diameter (mm)	1.0 ± 0.4	1.1 ± 0.4	1.0 ± 0.4	0.7 ± 0.2	<0.001
VCA diameter (mm)	1.9 ± 0.6	2.0 ± 0.5	1.9 ± 0.6	1.5 ± 0.6	<0.001
VC length (mm)	12.9 ± 1.9	12.9 ± 1.9	12.8 ± 1.8	13.4 ± 1.9	0.19
VC-MTP angle (°)	33.05 ± 7.71	33.15 ± 7.86	32.58 ± 7.76	34.14 ± 6.57	0.44
PVC-vomerine crest distance (mm)	5.9 ± 3.9	6.4 ± 3.8	5.5 ± 4.1	4.2 ± 3.8	<0.001
VC-vomerine crest distance (mm)	13.6 ± 1.7	13.5 ± 1.6	13.4 ± 1.7	14.8 ± 1.4	<0.001
VC-FR distance (mm)	8.1 ± 2.3	7.1 ± 2.1	9.3 ± 2.0	9.8 ± 2.2	<0.001
SPF diameter (mm)	3.9 ± 0.8	3.8 ± 0.8	4.0 ± 0.9	4.0 ± 0.8	0.19

VC, vidian canal; Type 1, within the sphenoid corpus; Type 2, partially protruding into the sphenoid sinus; Type 3, totally protruding into the sphenoid sinus; VCP, posterior opening of the vidian canal; VCM, middle part of the vidian canal; VCA, anterior opening of the vidian canal; PVC, palatovaginal canal; FR, foramen rotundum; SPF, sphenopalatine foramen; MTP, middle turbinate posterior end. Mean ± standard deviation.

**Table 6:** The relationship between the morphometric measurements and the pterygoid process pneumatization.

Morphometric measurements	Pterygoid process pneumatization		*p*-Value
	Not exist	Exist	
	Mean ± SD	Mean ± SD	
VCP diameter (mm)	1.6 ± 0.5	1.5 ± 0.5	0.06
VCM diameter (mm)	1.1 ± 0.4	0.9 ± 0.4	<0.001
VCA diameter (mm)	2.0 ± 0.6	1.7 ± 0.6	<0.001
VC length (mm)	12.8 ± 1.9	13.0 ± 1.8	0.22
VC-MTP angle (°)	33.21 ± 7.80	32.79 ± 7.58	0.54
PVC-vomerine crest distance (mm)	6.4 ± 3.8	5.1 ± 4.1	<0.001
VC-vomerine crest distance (mm)	13.5 ± 1.6	13.7 ± 1.8	0.39
VC-FR distance (mm)	7.2 ± 2.0	9.6 ± 2.1	<0.001
SPF width (mm)	3.9 ± 0.8	3.9 ± 0.8	0.37

VCP, posterior opening of the vidian canal; VCM, middle part of the vidian canal; VCA, anterior opening of the vidian canal; VC, vidian canal; PVC, palatovaginal canal; FR, foramen rotundum; SPF, sphenopalatine foramen; MTP, middle turbinate posterior end. Mean ± standard deviation.

## Discussion

Since the surgical approach to the PPF is difficult, the intraoperative complications such as excessive bleeding and nerve injury can occur. The vidian nerve, which is formed by deep (sympathetic fibers) and greater (parasympathetic fibers) petrosal nerve, courses through the VC that is an important landmark during endoscopic access to the PPF.^5^ Especially, for treating the resistant cases of chronic vasomotor rhinitis, the vidian neurectomy interrupts cholinergic innervation of the nasal mucosa by parasympathetic fibers.^6^ In previous studies, the endoscopic surgical approaches to the VC were classified as transsphenoidal (Type I) that transected the vidian nerve inside the sphenoid sinus and transnasal (Type II) that is performed in the anterior wall of the sphenoid sinus.^7^ Type I had its advantages shorter operating time, less risk of injury and reduced bleeding. The patients with no intrasphenoid septum, the VC canal corpus Type 2 and 3, the presence of the PPP, the greater distance between VC and FR and the VC not in extreme lateralized position were suitable for Type I approach. In other cases, Type II approach was mostly chosen.^4^, ^5^ The smaller angle between the posterior end of the middle turbinate and the lateral margin of the anterior opening of the VC, no extreme septum deviation, the presence of the gap between the sphenoid process of the palatine bone and the sphenoid bone just medial to the VC significantly increased the success rate of Type II approach.^5^, ^6^, ^7^ The VC corpus types and the PPP which affects the morphometric parameters should be evaluated on preoperative CT scan to avoid iatrogenic injury during endoscopic surgery.^4^

We observed the VC corpus types and the presence of the PPP with a correlation between them. The first aim of our study was to show the relationship of them with the morphometric measurements and the variations of the surrounding sphenoid structures. In previous studies, Bidarkotimath et al., Yeh et al., Chen et al. and Cankal et al. reported the prevalence of the VC Type 1 (within the sphenoid corpus) as 67%, 50.8%, 55%, 54%; Type 2 (partially protruding into the sphenoid sinus) as 22%, 39.8%, 31%, 36%; Type 3 (totally protruding into the sphenoid sinus with a stalk) as 11%, 9.4%, 14%, 10%, respectively.^4^, ^8^, ^9^, ^10^ In this study, we classified the VC variants as Type 1, 55.6% (278/500); Type 2, 34.8% (174/500); Type 3, 9.6% (48/500). Consequently, the VC Type 3 constitutes a minority while the VC Type 1 makes up half of the canals as similar to previous studies. The VC Type 1 that is embedded inside the sphenoid corpus inhibits the visualization of the VC in both surgical approaches. In this case, if there is no gap between the pterygoid process and the sphenoid process of the palatine bone, one of them is partially removed by transnasal approach.^5^, ^7^ On the other hand, the prevalence of the PPP was determined as 39.2% (196/500), similar to previous studies results that ranged between 19% and 43.6%.^4^, ^11^, ^12^ Also, we reported that the prevalence of the PPP that facilitates the Type I approach were significantly higher in VC Type 2 (72.4%) and Type 3 (95.8%) as shown in Table 3 (*p* < 0.001).

In the literature, the most common type of pneumatization of the sphenoid sinus was found as the sellar type.^5^, ^13^ We also observed the pattern of the sphenoid pneumatization and determined as the sellar type (67%). The sellar type pneumatization facilitates all of the endoscopic approaches but, the conchal (nonpneumatized) sphenoid which was found as 1% in our study is a relative contraindication for both types. The intrasphenoid septum is usually deviated and mostly attached to one side (ICAS or OCS).^5^ Fernandez-Miranda et al. reported that the prevalence of only one midline sphenoid septum was 87% and the ICAS was 13%.^14^ We reported that the number of the ICAS was 142 (28.4%), the OCS was 72 (14.4%) and the septum between the VC and vomerine crest was 66 (13.2%). According to Table 3, Table 4, the prevalence of the ICAS and the septum between the VC and vomerine crest were positively correlated with the VC protrusion and the presence of the PPP (*p* < 0.001), but the prevalence of the OCS was not affected. In this respect, our study is the first reporting that the presence of the ICAS and the intrasphenoid septum between VC and vomerine crest is associated with the VC protrusion and the PPP. Specifically, the presence of the intrasphenoid septum between VC and vomerine crest can inhibit visualization during surgery. Removal of the septum is time-consuming and can cause skull base penetration and injury to the internal carotid artery even though the VC protrusion and PPP exist.^4^ In addition, we analyzed that the VC is mostly located medial to the MPP and is not affected by the VC protrusion and the PPP in Table 3, Table 4 (*p* = 0.06, *p* = 0.16). Also, we found that there was no relationship between the septum deviation and other parameters. The medial location of the VC and no septum deviation facilitates the transnasal approach.

In the literature, the measured mean distances from vomerine crest to VC ranged between 12 and 16 mm and to PVC ranged from 8 to 11 mm, respectively.^4^, ^8^, ^15^ Also, the mean distances between FR and VC were measured as ranging between 4 and 8.5 mm.^3^, ^4^, ^8^, ^15^, ^16^ In our study, we measured the mean distance from vomerine crest to VC and to PVC as 13.6 ± 1.7 mm and 5.9 ± 3.9 mm, respectively. The mean FR-VC distance was measured as 8.1 ± 2.3 mm. According to Table 5, Table 6, the VC protrusion altered the VC-FR and VC-PVC distances which were larger in VC Type 2 and 3 (*p* < 0.001). Also, these distances were affected as increased with the presence of the PPP (*p* < 0.001). Yeh et al., Hewaidi et al., Vescan et al. and Citardi et al. reported a positive correlation between the PPP and the VC protrusion which increased the distance between VC and FR was similar with our study results that can provide a good surgical guide in both endoscopic approaches.^4^, ^11^, ^17^, ^18^ However, the smaller VC-FR and VC-PVC distances in VC Type 1 which had no protrusion into the sphenoid sinus can cause neurovascular complications that should be kept in mind by the surgeons. In addition, the PPP usually causes protrusion of the surrounding sphenoid structures into the sinus.^4^ So, during transsphenoidal surgery the surgeon should be careful to avoid the increased risk of neurovascular injury.

We reported that the measured mean diameters of the middle part, anterior and posterior openings of the VC were 1.0 ± 0.4 mm, 1.9 ± 0.6 mm and 1.6 ± 0.5 mm similar to the previous studies, respectively.^3^, ^8^, ^10^ Also, we found the mean length of the VC as 12.9 ± 1.9 mm while other studies reported values ranging from 12.5 to 17 mm.^8^, ^10^ Mato et al. reported that during the transnasal approach, the amount of bony drilling on the inferomedial surface of the VC depends on the length, protrusion of the VC and pneumatization degree of the pterygoid process. So, these data can guide a safe procedure to the anterior genu of the petrous ICA.^19^ But in this study we found that the length of the VC and the diameter of the posterior opening of the VC were not statistically related with the type of the VC and the degree of the PPP as seen in Table 5, Table 6. The mean diameters of the middle part and anterior opening of the VC were smaller in VC Type 3 and the VC with PPP (*p* < 0.001).

On the other hand, the anterior opening of the VC was located above and 4–6 mm posterolateral to the inferior margin of the SPF that is used in transnasal approach.^20^ Hwang et al. reported that the VC-SPF horizontal distance was positively, but the diameter of the SPF was negatively correlated with the VC-FR distance and measured the mean diameter of the SPF as 5.3 ± 1.3 mm on the three-dimensional reconstruction of the CT scans.^3^ We measured the mean diameter of the SPF as 3.9 ± 0.8 mm but, we reported that the diameter of the SPF was not correlated with the VC-FR distance, the VC corpus types and the PPP as seen in Table 5 and 6 (*p* = 0.19, *p* = 0.27).

The lateral margin of the anterior opening of the VC is usually located at the superolateral level of the posterior end of the middle turbinate; the smaller angle between them increases the success rate of the operations.^5^ Liu et al. measured the average value of that angle and compared to the operating success rates. They reported that the mean angle from axial and coronal CT imaging were 30.2 ± 4.9° and 26.4 ± 9.1° in the successful group, but the values were 33.8 ± 4.8° and 44.3 ± 8.1° in the failed group, respectively.^6^ In our study, we measured this angle as 33.05 ± 7.71° and found that the angle value was not affected by the VC protrusion and the PPP as shown in Table 5, Table 6.

This study has some limitations. Unapparent margins of the PPF complicate selection of suitable planes for measurements. CT imaging may not represent the true plane that is compatible when encountered during endoscopic surgery. Recruitment of the higher number of patients and patients from different races may give researchers more comprehensive results.

## Conclusion

In this study, we examined the VC in all respects and reported that the ICAS, the septum between the vidian canal and the vomerine crest, the VC corpus types, the VC-FR and the VC-PVC distances were affected by the presence of the PPP which play a key role in endoscopic surgery. But, we found that the presence of the PPP did not alter the distance from the vomerine crest to the VC and the location of the VC related to MPP. So, the PPP can have an influence on the configuration of the vidian canal but not on the position of it. Precise knowledge of the radiologic anatomy of the pterygopalatine fossa may be essential for diagnosing vidian nerve pathology and choosing an appropriate endoscopic approach and side of the surgical intervention. As a result, the surgical complications associated with an endoscopic vidian neurectomy can be decreased.

## Conflicts of interest

The authors declare no conflicts of interest.
